# Particle Swarm Optimization and Uncertainty Assessment in Inverse Problems

**DOI:** 10.3390/e20020096

**Published:** 2018-01-30

**Authors:** José L. G. Pallero, María Zulima Fernández-Muñiz, Ana Cernea, Óscar Álvarez-Machancoses, Luis Mariano Pedruelo-González, Sylvain Bonvalot, Juan Luis Fernández-Martínez

**Affiliations:** 1 ETSI en Topografía, Geodesia y Cartografía, Universidad Politécnica de Madrid, 28031 Madrid, Spain; 2 Grupo de Problemas Inversos, Optimización y Aprendizaje Automático, Departamento de Matemáticas, Universidad de Oviedo, 33007 Oviedo, Spain; 3 Laboratoire GET, Université de Toulouse, CNRS, IRD, CNES, 31400 Toulouse, France; 4 Bureau Gravimétrique International (BGI), 31401 Toulouse, France

**Keywords:** inverse problems, nonlinear inversion, noise and regularization, model reduction, uncertainty analysis, particle swarm optimization (PSO)

## Abstract

Most inverse problems in the industry (and particularly in geophysical exploration) are highly underdetermined because the number of model parameters too high to achieve accurate data predictions and because the sampling of the data space is scarce and incomplete; it is always affected by different kinds of noise. Additionally, the physics of the forward problem is a simplification of the reality. All these facts result in that the inverse problem solution is not unique; that is, there are different inverse solutions (called equivalent), compatible with the prior information that fits the observed data within similar error bounds. In the case of nonlinear inverse problems, these equivalent models are located in disconnected flat curvilinear valleys of the cost-function topography. The uncertainty analysis consists of obtaining a representation of this complex topography via different sampling methodologies. In this paper, we focus on the use of a particle swarm optimization (PSO) algorithm to sample the region of equivalence in nonlinear inverse problems. Although this methodology has a general purpose, we show its application for the uncertainty assessment of the solution of a geophysical problem concerning gravity inversion in sedimentary basins, showing that it is possible to efficiently perform this task in a sampling-while-optimizing mode. Particularly, we explain how to use and analyze the geophysical models sampled by exploratory PSO family members to infer different descriptors of nonlinear uncertainty.

## 1. Introduction

Inverse problems belong to the class of ill-posed problems (see [1]); that is, either the solution does not exist, it is not unique, or it is unstable, because it does not depend continuously on the observed data. Uncertainty in discrete inverse problems is due to noise in data, incomplete data coverage, modeling assumptions and numerical approximations, among other causes. Traditionally, the ill-posed character of nonlinear inverse problems has been approached via local optimization methods combined with regularization techniques, but the uncertainty appraisal that is done using linearization techniques is only accurate in the neighborhood of the inverse solution (see [2]).

Typical inverse problems in geophysics are, for example, the determination of the mass distribution in the earth using gravity measurements taken at the terrain surface (see for instance [3]), finding the slowness distribution of a geological medium (see for instance [4]), the determination of the reservoir permeability field from historical data (see for instance [5]), and the characterization of the geoelectrical structure of the subsurface via resistivity imaging (see for instance [6]), to cite some of the examples our research group has investigated.

Discrete inverse problems consist of solving the following set of equations:

(1) $$F\left(m\right)=d^{\mathrm{obs}}$$

where m∈M is the discrete inverse model; $d^{\mathrm{obs}}\in D$ is the observed data array, which belongs to the data space D; and F:M⟶D represents the forward problem functional. A particular model m is expressed by its coordinates in a given basis set of the model space as $m=\left\{m_{1},m_{2},\cdots ,m_{n}\right\}$. Analogously, the observed data can be written as $d^{\mathrm{obs}}=\left\{d_{1},d_{2},\cdots ,d_{s}\right\}$. The model and data spaces are Euclidean spaces, to be able to measure distances between predictions and observations as well as distances between models with respect to a reference. If the forward operator F corresponds to a linear application $F:R^{n}⟶R^{s}$, the inverse problem is linear. Otherwise, the inverse problem is nonlinear; that is, there is a nonlinear dependence of F on m.

The idea of solving an inverse problem by only determining one *best* model requires revision (see [7]). Additionally, inverse problems are a special kind of optimization problem, because the observed data enters into the cost function. Therefore, as a result of the effect of noise in data, the global optimum of the cost function is never the model that has generated the observed data, in case this model exists (see [8] for details).

In mathematical terms, the uncertainty analysis consists of finding the family $M_{\mathrm{tol}}$ of plausible models m that fit the observed data $d^{\mathrm{obs}}$ within a given error tolerance «tol»:

(2) $$m\in M_{\mathrm{tol}}\;:{∥F\left(m\right)-d^{\mathrm{obs}}∥}_{p}\leq \mathrm{tol}$$

where *p* is the norm used in the data space, usually the $L_{2}$ norm (p=2). Therefore, the uncertainty analysis is related to the cost-function topography (see [2]). In the linear case, the misfit function is valley-shaped, elongated in the directions corresponding to the smaller singular values of F. In the case of nonlinear problems, this topography persists (up to a first-order approximation) in the directions corresponding to the smaller singular values of the Jacobian of F. These valleys adopt a curvilinear shape, which is frequently named a banana or croissant shape (see [2]), because of the dependence of the Jacobian on the discrete models m. Additionally, several local minima might also coexist in the misfit landscape and are related to the nonlinear effects ignored by the Gauss–Newton approximation to the Hessian. This fact also explains why linearization techniques fail in addressing the uncertainty analysis of nonlinear inverse problems.

The structure of this paper is as follows: Section 2 briefly describes the effect of noise in data and that of the regularization for linear and nonlinear inverse problems (see [2,8,9,10] for a deep understanding of the topic). This analysis is important for understanding that uncertainty assessment via linearization techniques always leads to incorrect results, as these techniques are not able to analyze the nonlinear uncertainty. Section 3 is a brief presentation of the different particle swarm optimization (PSO) family members. Section 4 shows the application of this methodology to gravity inversion in sedimentary basins. Section 5 briefly talks about the use of model reduction techniques and their combination with PSO to address the solution and model appraisal in high-dimensional inverse problems. Finally, Section 6 outlines the main conclusions of this paper, aiming to convince practitioners that uncertainty analysis is a compulsory step in inversion that can be efficiently done via exploratory global optimization methods.

## 2. The Effect of Noise and Tikhonov’s Regularization in Inverse Problems

The concept of noise is intimately related to the quality of the solution found via inversion, as a poor understanding of its effect may cause the signal, the noise and the modeling errors to be confused, introducing very important biases. The difference between noise and modeling errors is also in some situations very difficult to find. Typically, modeling errors are confounded with colored noise, while measurement noise has a random structure. In the case of nonlinear inverse problems, we denote by $c_{p}\left(m\right)$ the perturbed cost function due to the influence of noise δd in data; then we have

(3) $$\begin{array}{cc} c_{p}\left(m\right) & =∥F\left(m\right)-d^{\mathrm{obs}}{∥}_{2}^{2}={∥F\left(m\right)-\left(d^{\mathrm{true}}-\delta d\right)∥}_{2}^{2}\\ & =∥F\left(m\right)-d^{\mathrm{true}}{∥}_{2}^{2}+{∥\delta d∥}_{2}^{2}-2\delta d^{T}\left[F\left(m\right)-d^{\mathrm{true}}\right]\\ & =c\left(m\right)-2\delta d^{T}\left[F\left(m\right)-d^{\mathrm{true}}\right]+{∥\delta d∥}_{2}^{2} \end{array}$$

where $d^{\mathrm{obs}}=d^{\mathrm{true}}+\delta d$ is the sum of the true observations plus the noise. The minimum of $c_{p}\left(m\right)$ is achieved in the model $m_{0}$, where the gradient of the cost function is null: $\nabla c_{p}\left(m_{0}\right)=0$. ${JF}_{m_{0}}$ being the Jacobian of F in $m_{0}$, the stationary condition implies the following (see [10] for details):

(4) $$\nabla c\left(m_{0}\right)=2{JF}_{m_{0}}^{T}\delta d$$

instead of $\nabla c\left(m_{0}\right)=0$, as would happen in the noise-free case. In the presence of data noise, the minimum of the perturbed cost function will never correspond to the minimum in the noise-free case. Therefore, uncertainty analysis is always needed to assess the presence of other plausible scenarios, which could be located in different low-misfit basins.

Additionally, using mathematical analysis, it has been proved (see [2,9,10]) that noise deforms the cost-function topography (and particularly the region of equivalence) in linear and nonlinear inverse problems. The method consists of establishing the condition for which the equivalence region for a given tolerance value has the same spatial extent under the presence of data noise with respect to the noise-free case. In the case of linear inverse problems, this deformation is homogeneous, whereas in the case of nonlinear inverse problems, it depends on the misfit region, decreasing the size of the regions with lower misfits and eventually increasing the regions of medium–high misfits. In this way, finding a model with a lower misfit becomes a harder task, but locating the region of medium misfits using search methods might become simpler. This fact has been proved via numerical experimentation in synthetic cases (see [5]).

The following two-dimensional (2D) synthetic example (presented in [10]) serves to show the influence of noise in nonlinear inverse problems. The data predictions in the different $x_{k}$ points are given by

(5) $$y_{k}=\alpha \left(1-e^{-\beta x_{k}}\right)+\delta {}_{k}$$

where $\delta {}_{k}$ represents the effect of the observational noise. The inverse problem consists of identifying the parameters (α,β) from a set of data points $\left\{\left(x_{1},y_{2}\right),\left(x_{2},y_{2}\right),\cdots ,\left(x_{s},y_{s}\right)\right\}$ that have been generated using $\left(\alpha_{t},\beta_{t}\right)=\left(20,0.1\right)$ as the true model. We have added two different levels of white noise to the observations ($\delta_{1}\to N\left(0,0.05\right)$ and $\delta_{2}\to N\left(0,0.075\right)$), and the nonlinear parameter identification problem has been solved using the Gauss–Newton technique with $m_{0}=\left(\alpha_{0},\beta_{0}\right)=\left(15,0.06\right)$ as the initial guess.

**Figure 1:**
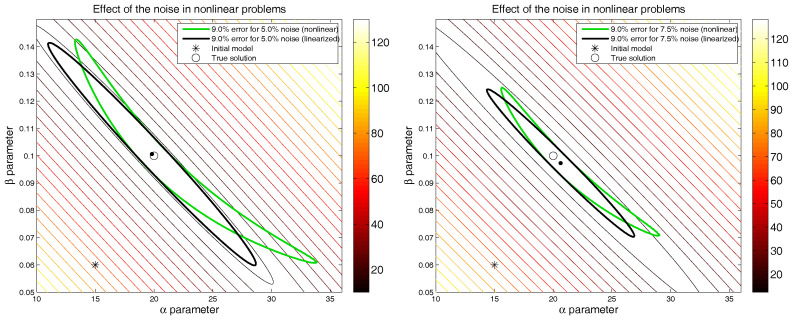
Synthetic numerical example. The left figure shows the effect of noise for a nonlinear problem without and with white noise, N(0,0.05). Right figure shows the case with white noise, N(0,0.075).

Figure 1 shows the results obtained for both inversions compared to the true model. The nonlinear (green line) and the linearized (black line) equivalent regions of 9% relative error are also shown. Some interesting observations can be made:

1. The typical banana shape of the region of equivalence in nonlinear inverse problems can be observed (see [2]). Additionally, the hyperquadric corresponding to the linearized region of equivalence (computed in the Gauss–Newton solution) represents the nonlinear uncertainty region only locally. The uncertainty assessment performed by taking into account the linearized region of equivalence will produce wrong results, particularly in highly nonlinear problems with complex cost-function topographies, as the linearized region contains models that do not belong to the nonlinear region of equivalence and ignores others that do belong to this region.
2. The nonlinear and linearized equivalence regions of 9% relative misfit decrease in size as the noise level increases.
3. The solution in the noisy case might move further away from the true solution as the noise level increases, particularly in the absence of the regularization term. The procedure might converge to a solution located in another disconnected basin of the cost-function topography, depending on the reference model that is used. This also depends on the statistical distribution of the noise that is added to perturb the observed data.

A similar analysis can be done to understand the effect of the regularization. As has already been explained, inverse problems are unstable, and this fact provokes dramatic perturbations of their solution when they are solved via local optimization techniques that build a numerical approximation of the inverse operator. To stabilize their solution, different types of regularization have been introduced (see [11,12,13]). Tikhonov and Arsenin introduced the concept of a *correctness set*, for which the inverse operator is continuous and the inverse problem becomes well-posed. They also proved that it is possible to achieve a stable pseudo-solution (also called quasi-solution) by performing minimization in the correctness set (see [14]). A way of constructing this set is to approximate the original ill-posed problem by a class of well-posed regularized problems of the following type: $m_{\varepsilon }=F_{\varepsilon }^{-1}$, where ε>0 is the regularization parameter, designed to approach zero. The simplest way to define $F_{\varepsilon }$ is to introduce in the regularized misfit function $c_{\varepsilon }\left(m\right)$ a penalization term that takes into account the distance to a reference model $m^{\mathrm{ref}}$:

(6) $$c_{p}\left(m\right)=∥F\left(m\right)-d^{\mathrm{obs}}{∥}_{p}+\varepsilon {∥m-m^{\mathrm{ref}}∥}_{q}$$

where *p* and *q* represent respectively the norms adopted in the data and model spaces (usually the Euclidean norm).

The regularization induces a deformation of the linearized region of equivalence for the regularized inverse problem. This deformation is non-homogeneous and anisotropic; that is, it affects differently each of the principal directions of the region of equivalence, bounding this region more intensely along the axes with higher uncertainty. Tikhonov’s regularization might also be responsible for the introduction of local minima in the cost-function topography (see [2]).

**Figure 2:**
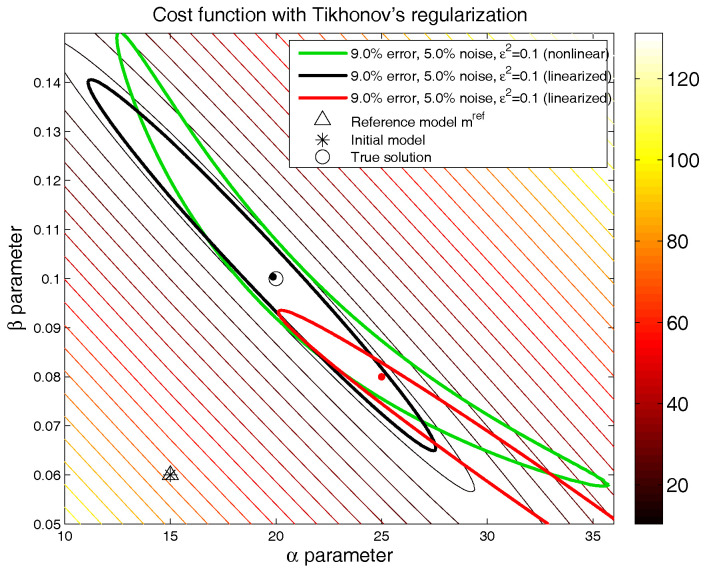
Synthetic numerical example. Comparison of the nonlinear and linearized regions of equivalence with Tikhonov’s regularization for two different models located on the nonlinear region of equivalence; noise: δ→N(0,0.05), regularization parameter: ε=0.1; $m^{\mathrm{ref}}=\left(15,0.6\right)$.

Figure 2 presents, for the above-mentioned synthetic numerical example, the linearized equivalent region around two different models, which belongs to the nonlinear equivalent region of 9% relative misfit (green line): $m_{0}=\left(20,0.1\right)$ (black line) and $m_{0}=\left(25,0.08\right)$ (red line). The parameters used in this numerical experiment were the following: the initial guess to compute the inversion coincided with the reference model adopted in the regularization, $m^{\mathrm{ref}}=\left(15,0.06\right)$; the damping parameter was $\varepsilon^{2}=0.1$; and a noise term δ→N(0,0.05) was added to the synthetic data. As was previously outlined, the linearized region of equivalence only represents the nonlinear region of equivalence locally. Additionally, this analysis depends strongly on the model that it is used to calculate the Jacobian.

## 3. Sampling Uncertainty via the Particle Swarm Optimization Algorithm

The solution of nonlinear problems via local optimization techniques provides geophysical models that strongly depend on the initial model that is adopted and on the kind of regularization that it is used. Particularly, the reference model used in the regularization term has a crucial effect in the inversion. These techniques are used in combination with linear uncertainty analysis to assess the quality of the solution that has been obtained. Nevertheless, as has been shown in Figure 1 and Figure 2, the linearized uncertainty region (or linearized region of equivalence) only partially accounts for the true nonlinear uncertainty. Other possibilities concern the use of Monte Carlo methods that randomly sample the nonlinear equivalence region following a Bayesian approach (see [15]). However, these methods are not feasible for high dimensions because of the curse of dimensionality (see [16]).

The point of view followed by Tarantola [7] does not consist of the solution display supplemented by a technical description of the corresponding uncertainties, but rather by a display of as many solutions as possible consistent with the prior information and the observed data. In fact, the uncertainty analysis in inverse problems consists of sampling low-misfit elongated curvilinear valleys of the data-prediction cost function to obtain a representative set of samples from the nonlinear uncertainty region (see [2,8] for details).

Global optimization methods are a good option to sample the set of equivalent models when they are used in exploratory form. This is the case of binary genetic algorithms with a high mutation rate (see [17]) and also for different members of the PSO family (see [18]). These methods do not suffer from instability as they do not solve the inverse problem as an optimization problem, but as a search or sampling problem, using only the forward model to calculate the misfit of the different sampled models. These methods do not construct an approximation of the inverse operator. Therefore, the regularization term and the initial model are not needed. Additionally, in the case of costly forward solutions, it is crucial for these algorithms to be able to sample the region of uncertainty with a limited number of forward solves. PSO shows a good trade-off between exploration and exploitation.

### 3.1. The PSO Family

PSO (see [19]) is a global optimization algorithm that is inspired by the behavior of bird flocks and fish schools searching for food, for which a swarm of particles examines the space of solutions in order to optimize a given cost function. The PSO flowchart for optimization problems is as follows: the PSO particles (geophysical model parameters in our case) are represented by vectors of length equal to the optimization problem unknowns. The initial set of particles is initialized using random positions $x^{0}$ and null velocities $v^{0}$, and the cost function (Equation (3)) is evaluated to compute the misfit (prediction error) of each particle in the swarm. As iterations progress, the position and velocity of each is updated as a function of its fitness and the corresponding values of its neighbors. At time-step k+1, the PSO algorithm updates the individuals’ positions $x^{k+1}$ and velocities $v^{k+1}$ as follows:

(7) $$\left\{\begin{array}{c} v_{i}^{k+1}=\omega v_{i}^{k}+\phi_{1}\left(g^{k}-x_{i}^{k}\right)+\phi_{2}\left(l_{i}^{k}-x_{i}^{k}\right)\\ x_{i}^{k+1}=x_{i}^{k}+v_{i}^{k+1} \end{array}\right.$$

In Equation (7), $g^{k}$ is the global best position in the whole swarm, $l_{i}^{k}$ is the *i*th particle’s best position, $\phi_{1}=\;r_{1}a_{g}$ and $\phi_{2}=r_{2}a_{l}$ are random accelerations (global and local, respectively), and ω is the inertia weight (a real constant); $r_{1}$ and $r_{2}$ are random variables uniformly distributed in (0,1) to weight the global and local acceleration constants $a_{g}$ and $a_{l}$, but other statistical distributions could also be used. The PSO parameter tuning consists of providing proper values for the inertia constant ω and for the local and global accelerations $a_{g}$ and $a_{l}$, to achieve the exploration of the low-misfit regions of the cost function.

PSO can be interpreted as a double stochastic gradient in the model space. It is a special case of the generalized PSO (GPSO) algorithm (see [20]), which was developed following the analogy of a damped mass–spring scheme with unit mass, damping factor 1−ω, and total stiffness constant $\phi =\phi_{1}+\phi_{2}$. A full family of particle swarm optimizers (CC-PSO, CP-PSO, PP-PSO and RR-PSO) was also developed on the basis of this analogy using different discretizations for the velocity and the accelerations (see [21,22,23] for details). The stochastic stability analysis of the PSO trajectories was performed in [20,21,24] and served to establish the relationship between PSO convergence and the first- and second-order stability of the trajectories of the particles considered as stochastic processes. Suitable PSO parameters ($\omega ,a_{g}$ and $a_{l}$) are located in the neighborhood of the upper border of the second-order stability region for each member of the PSO family. This result is also true for any statistical distribution of the PSO parameters (see [25]). The cloud versions of these algorithms are based on this mathematical analysis (see [18]). In the cloud versions, no parameter tuning (inertia, local and global accelerations) is needed, as each particle has its own parameters that are randomly selected from a set of PSO parameters that are located in the neighborhood of the upper limit of their second-order stability regions. Additionally, in the cloud design, each particle has its corresponding time-step Δt value. The exploration of the search space increases when Δt is greater than 1.0. Conversely, the algorithm freezes the solution found when Δt is decreased to values lower than 1.0.

The case of the RR-PSO algorithm is slightly different, as it has been numerically shown that good parameter sets are located along the line $\bar{\phi }=3\left(\omega -3/2\right)$, mainly for inertia values ω>2 (see [22]). This straight line does not depend on the cost function and remains invariant when the number of parameters increases. Additionally, this line is located in a region of medium attenuation and very high frequency for the particle trajectories. This feature confers to RR-PSO a good equilibrium between exploration and exploitation, allowing for an efficient and exploratory search.

The numerical experiments using different well-known benchmark functions have shown that the best-performing algorithm of the PSO family is RR-PSO (see [22]). CP-PSO is the most exploratory family member. Therefore, it is an interesting choice for performing nonlinear uncertainty analysis and exploring the cost-function topography efficiently. PP-PSO has the same velocity update as GPSO, but the positions of the particles are in time *t* instead of t+1. PP-PSO has a more exploratory character than GPSO but a lower convergence rate. Finally, CC-PSO has showed the fastest convergence rate. In any case, it is always convenient to check their results with a new problem to select the more appropriate version. In general terms, all the PSO family members provide excellent results as long as the parameter tuning is correct, and this is related to the second-order stability of the trajectories. This way, no fancy or strange mechanisms are needed to avoid the misunderstood phenomenon of premature convergence. All these mathematical results make PSO a very unique algorithm, different in any case from other heuristic approaches (see [26,27]). Additionally, the PSO family members are able to provide a set of representative samples of the nonlinear region of equivalence, which can be used to infer an approximate posterior of the model parameters in nonlinear inverse problems much faster than Monte Carlo methods and much more realistically than linear analysis techniques combined with local optimization methods.

### 3.2. Application of the PSO Family to Geophysical Inversion

As an example of this methodology, we show the application to gravity inversion in geophysical exploration. The use of PSO in applied geophysics was introduced in [18,28,29,30]. Particularly, Fernández-Martínez et al. presented in [18] the application of GPSO, CC-PSO and CP-PSO to the solution and appraisal of a 1D-DC (Vertical Electric Sounding, VES) resistivity inverse problem, successfully comparing the results with Metropolis–Hastings. In gravity inversion, PSO has been used in synthetic examples to compare with other global and local search methods without performing an uncertainty assessment (see [31,32,33]). This way of proceeding is not correct, as we have already conveniently explained. PSO was used for the first time in gravity inversion applied to basement depth determination in sedimentary basins in [3,34].

Prior to the PSO sampling, we need to specify the lower and upper bounds of the parameter search space, the number of particles and iterations, and the basis set in which the sampling will be performed. The search space should be designed according to the geological prior knowledge that we have at disposal for the model parameters; that is, the algorithm will explore the portion of the cost function bounded by the search limits. In some problems, the observations themselves can be used to estimate such limits. For example, the upper and lower depth limits of the search space in basement depth gravity inversion can be computed using Bouguer’s plate formulation (see [3,34]). The search space design can be used also to impose absolute constraints on some parameters and reduce their uncertainty and the trade-offs with others. This information can come from boreholes or from other geophysical techniques. The use of all possible sources of prior information is a key concept in inverse problems. Nevertheless, all these pieces of information must be carefully examined to avoid inconsistencies (see [7]).

The number of particles depends on the computational cost needed to solve any individual forward problem (see [5]). Nevertheless, when a maximum number of forward solves can be achieved, it is more convenient to increase the number of iterations than the number of particles in each iteration. Despite this, only a fast numerical experimentation can help to establish the optimum trade-off between these two parameters.

Regarding the stopping criteria in this sampling procedure, the PSO algorithm is designed to finish by iterations. Nevertheless, the PSO design has to avoid the collapse of the swarm towards a unique particle that would introduce a bias in the posterior analysis. For that reason, it is very important to monitor the dispersion of the swarm with iterations. In each iteration, the dispersion can be calculated through the median distance between the particles and their center of gravity with respect to the median distance in the first iteration, where the dispersion is considered to be 100%. In the case that this collapse occurs, there are several alternatives to avoid the oversampling of the misfit region: (1) to stop the sampling and finish the PSO execution; (2) to increase the exploration by increasing the time-step or introducing repulsive forces (see [18]); (3) to consider in the posterior analysis all the collapsed particles as a unique posterior model (see [5]). It is important to understand that the PSO sampling-while-optimizing procedure is just a numerical approximation of the Bayesian posterior. Finally, the selection of the basis set to achieve the sampling is explained at the end of the paper.

## 4. Uncertainty Analysis Using the PSO Sampling; Application in Gravimetry

Uncertainty analysis consists of obtaining a representative sample of the region $M_{\mathrm{tol}}$ to perform posterior statistics by simple counting, so as to adopt risk decisions. PSO obtains a collection of models from the nonlinear uncertainty region. All these models explain the observations with an error level lower than the prescribed tolerance (see Equation (2)). The rest (those that do not fulfill the misfit criterion) come from different regions of higher misfit. Therefore, the use of a global search algorithm in their exploratory form provides a more general overview about the equivalent solutions of the inverse problem than local optimization methods. According to Tarantola (see [7]) and Scales and Snieder (see [35]), this collection of models can be considered itself the inverse problem solution, as all of the models explain the observations at the prescribed error level and are prior compatible. The model with minimum misfit is another model in this set, and, as has been explained in the section concerning the effect of noise, it is the only model that could not have generated the observed data. Additionally, it can be shown via synthetic experiments that the true model will belong to the nonlinear region of uncertainty and will have a higher misfit. This fact suggests the need of performing a correct uncertainty analysis.

### 4.1. Gravity Inversion in Sedimentary Basins

Estimation, using surface gravity measurements, of the vertical separation interface between two media that have different densities (where the upper, embedded in the lower, has a density $\rho_{u}<\rho_{l}$, as is common in sedimentary basins) is a nonlinear inverse problem. Gravity inversion is a classical tool, widely used in geophysics, oil and gas exploration, hydrogeology, glaciology, and so forth (see [36,37,38,39,40]). The gravity inverse problem, as a potential field method, has an inherent non-uniqueness in the solution, which leads to an infinite number of mass distributions generating the same gravitational signal (see, e.g., [14,41,42,43]). It is therefore mandatory to introduce some kind of constraints and take into account external information (as borehole data, seismic profile information, contrasted prior models, etc.) in order to bound the set of plausible solutions.

Once the gravity is observed and corrected (specific gravimeter corrections, time-dependent signals, atmospheric effects, etc.), the measurements are transformed into anomalies Δg through the classic and well-known free-air, Bouguer’s plate and terrain reductions, and the subtraction of the normal gravity field signal (see [38,40], for example). These values, called complete Bouguer’s anomalies, contain information about the crust–mantle interface and the mass distribution in a wide range of depths for the earth’s crust; thus their influence is considered as regional. Although some sedimentary basins whose area covers thousands of square kilometers exist, their depths are commonly small compared to the earth’s crust thickness; thus for a correct problem interpretation, it is necessary to isolate the part of Δg generated only by the sediments. This part is commonly called the local or residual anomaly, while the part of the signal due to the regional and deep mass distributions is known as the regional trend. These components can be isolated as a previous step or during the inversion process itself. A variety of methods exist to perform this task (see [44,45,46] for some examples).

Residual anomalies Δg on sedimentary basins usually have negative values, as the anomalous mass derived from a contrast density between the sediments and the basement has a negative sign: $\Delta g=\rho_{s}-\rho_{b}<0$. These anomalies are related to the basin depth (sediments layer thickness), and they constitute the fundamental observable for gravity inversion. This signal might have several contributions:

1. The anomaly due to the sediments themselves.
2. Observational noise, which is always present.
3. Modeling errors introduced by Bouguer’s anomaly correction, among others.
4. Modeling errors introduced by the regional trend suppression.

Noise and modeling errors introduced in points 2, 3 and 4 contribute to the lack of uniqueness in the solution and therefore to the uncertainty problem. These contributions should be properly taken into account in the interpretation of the results.

The most commonly used techniques for the solution of this inverse problem are based on local optimization methods (see [47], for example) or even the sequential solution of the forward problem (see [48]). Global search methods have also been proposed for this particular problem in [49], although the uncertainty assessment of the solution was not performed. For basin modeling, several alternatives have been suggested (for both 2D and three-dimensional (3D) models), such as, for example, its approximation as regular geometric bodies (see [50]), irregular polygons (see [51]), or the discretization in rectangles and prisms for 2D and 3D models, respectively (see [47,52]). Regarding the density contrast Δρ between the sediments and the basement, the nonlinear approach for the problem, in which the interface depths are the unknowns, imply that Δρ must be postulated as a constant value, or being variable with depth.

### 4.2. Argelès-Gazost Basin Gravity Inversion

**Figure 3:**
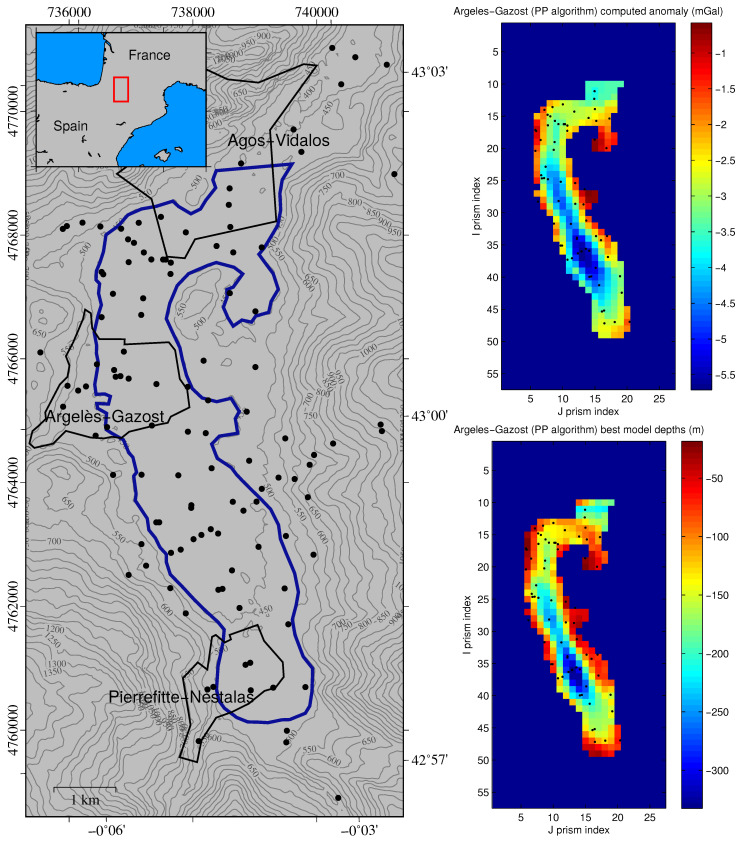
Left: Contour of the Argelès-Gazost basin (blue line) and observational points (black dots). Upper right: Computed gravity anomaly of the best model after the particle swarm optimization (PSO) execution. Lower right: Best depth model estimated via PSO (PP algorithm). Figure from [34].

As an example, we refer in this section to the gravity inverse problem in sedimentary basins in three dimensions [34]. In this work, the basement depth of the Argelès-Gazost basin, an ancient glacial valley located in the French Pyrenees, was determined via PSO using gravity anomalies measured on the terrain’s surface. Figure 3 (left) shows the approximate limits of the basin (blue line) and the points at which the gravity anomaly was measured (a total of 117 points, 69 of them located inside the basin limits). The basin was divided into 300 prisms of 225 m × 255 m in horizontal coordinates. The model parameters of the inverse problem were the depths of the basement in this grid. A density contrast between the sediments and the basement of $\Delta \rho =-600kg/m^{3}$ was used, and for the PSO execution (PP-PSO algorithm produced the best results) a swarm of 200 models and 200 iterations was employed; thus 40,000 forward models were solved, and the execution took less than 5 min on a personal computer (Intel Core i7-4800MQ 2.70 GHz running Matlab R2017a on a Debian GNU/Linux operating system). Figure 3 (right) shows the computed gravity anomaly (upper image) corresponding to the best model and the best model itself, that is, the basement depths (lower image). The best model had a relative error of 9.72%. The first step in the uncertainty analysis is the selection of the sampled models whose misfits are below a given error tolerance (working error bound); that is, the uncertainty analysis is done only with the PSO sampled models located in the low-misfit regions of the cost function. In this case, the error tolerance used was 15%, and PSO was able to sample 25,429 models within this region.

**Figure 4:**
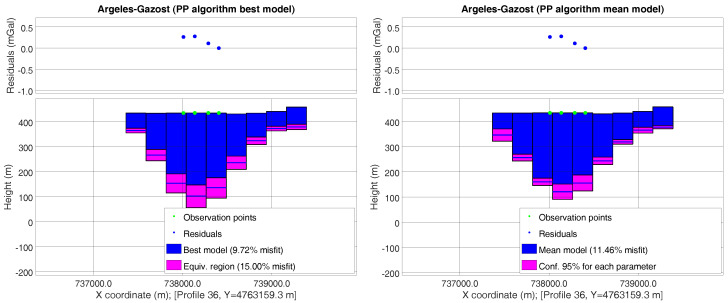
Left: West–East profile of the Argelès-Gazost basin containing the deepest point, belonging to the best particle swarm optimization (PSO) model (PP algorithm) and equivalent region of 15%. Right: Mean model corresponding to the same profile and confidence intervals of 95% for each parameter.

The first approach consisted of establishing a minimum–maximum range for each parameter, taking into account the geophysical models that have been sampled within the 15% nonlinear uncertainty region. Figure 4 (left) shows a West–East profile (that which includes the deepest point, where the profile number corresponds to the I prism index in Figure 3, right lower) of the Argelès-Gazost basin. It can be observed that the best PSO model had a misfit of 9.72%; the deepest point reached a depth from the surface of about 332.7m, and it belonged to a range of 288–379 m.

The second approach consisted of calculating the mean (or median) and standard deviation (or interquartile range) for each geophysical parameter, by averaging the above-mentioned geophysical models. This way, a “most-probable mean model” can be determined, together with some confidence intervals. Clearly, these results are approximate in nonlinear inversion, as the posterior distribution of the model parameters is typically non-Gaussian and/or multimodal. Figure 4 shows the best model found by PSO with the 15% nonlinear equivalence region (left) and the mean model with its 95% confidence interval (right). The misfit of the mean model (11.5%) was higher than that of the best model, but it was located inside the 15% tolerance region. The deepest point was located at 317 m below the surface, and its 95% confidence interval was 300–334 m. These are two different ways of visualizing the uncertainty.

**Figure 5:**
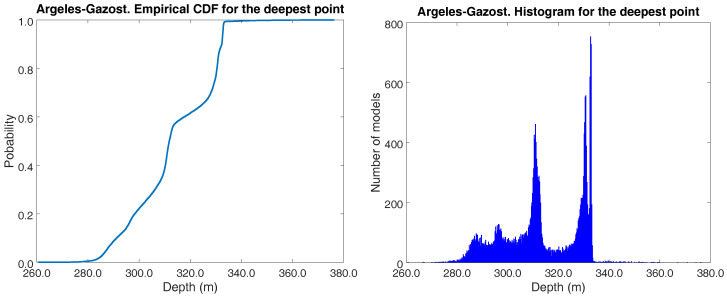
Argelès-Gazost basin. Left: Empirical cumulative density function corresponding to the deepest prism. Right: Absolute histogram for the deepest prism.

Additionally, different percentiles for the deepest point can be determined from the set of sampled models. Figure 5 (left) shows the empirical cumulative density function corresponding to the deepest prism in the Argelès-Gazost basin. The inferred parameter distribution is multimodal, as can be seen clearly in Figure 5 (right). This fact is an example of the complex cost-function landscape of this kind of inverse problem and the limited validity of a unique geophysical model found by local optimization. This information is crucial to assess risk.

## 5. PSO Combined with Model Reduction Techniques

PSO can be used for sampling using different basis sets. This fact depends on the number of model parameters of the inverse problem. It has to be understood that the number of parameters of the inverse and forward problems does not have to be the same, as model reduction techniques can be used for sampling purposes. In general terms, when the number of parameters is lower than 100, the sampling can be performed in the original basis set, although the curse of dimensionality indicates that it is impossible to efficiently sample a model space with more than 10 independent dimensions. Clearly, this is done by informing the search space. This strategy was used for instance in [3,30] using approximate solution methods. For very high dimensional inverse problems, it is compulsory to reduce the dimension by changing the basis set in which the sampling is performed. The reason is as follows: a highly underdetermined linear system transforms to an overdetermined linear problem if the number of basis terms in which the solution is found is smaller than the number of observed data points. Model reduction also serves to simplify the topography of the cost function and can be interpreted as a way of reducing uncertainty to the set of plausible models (see [53]).

The basis set can be established on the basis of a set of prior scenarios that are generated via conditional simulation. Then, their spectral basis can be established using principal component analysis (PCA) to avoid redundancy and reduce the dimension. This strategy was used in reservoir history matching (see [5]). The model reduction can also be based on the spectral decomposition of a model that is already located in the low-misfit region via local optimization methods. This strategy was followed in the electrical tomography problem in two dimensions (see [6]). In this case, the spectral basis set was established via the singular value decomposition (SVD), and the PSO sampling was performed in this reduced space. A high-frequency term can be added to the expansion to improve the quality of the sampling. In this case, it is recommended to have a reduced space whose dimension is lower than 10, which is the limit established by the curse of dimensionality in an isotropic space.

## 6. Conclusions

Inverse problems in the industry usually have a very underdetermined character. Particularly, in geophysical inversion, it is common to deal with data that is insufficient in quantity and is affected by noise, which together with the modeling and numerical errors cause the inverse problem solution to have an intrinsic uncertainty. Uncertainty always exists, and it is a synonymous of ambiguity; that is, the observed data never contain enough information to uniquely determine a true unique inverse solution. Nevertheless, practitioners tend to minimize the importance of uncertainty in inverse solutions, relying on the facts that (1) uncertainty always has a random structure that is impossible to be known; (2) regularization techniques provoke the vanishing of the equivalent solutions; and (3) linear analysis techniques provide a fairly good approximation of the real uncertainty. These three assumptions are false and might motivate that the decisions taken on the basis of this kind of analysis are erroneous, as a consequence inducing large economic losses.

Therefore, a proper nonlinear uncertainty analysis is always needed and should be understood in a very pragmatic way, as its final end is to be able to unravel plausible scenarios that were not and cannot be unravelled by the inversion. Global search methods offer a good alternative to Bayesian sampling methods when they are correctly designed. The most important feature is to be able to explore the nonlinear uncertainty region. We have shown some examples in gravimetry inversion, and we have referred to some of our previous work that used these methodologies. The final aim is of this paper is to convince practitioners that no further inversions without their corresponding uncertainty analysis should be accepted.
